# A rare case of sporadic metastatic colorectal cancer with glioblastoma multiforme: a challenging clinical scenario

**DOI:** 10.1093/jscr/rjae708

**Published:** 2025-02-17

**Authors:** Farah Awad, Firas Abdallah, Ahmad Alhalabieh, Imad Aljaafreh, Salam Naserallah, Elias Edward Lahham

**Affiliations:** Oncology Department, Augusta Victoria Hospital, East Jerusalem, Palestine; Faculty of Medicine, Al-Quds University, University Street, Abu Dis, Jerusalem District, 9700, Jerusalem, Palestine; Faculty of Medicine, Al-Quds University, University Street, Abu Dis, Jerusalem District, 9700, Jerusalem, Palestine; Faculty of Medicine, Al-Quds University, University Street, Abu Dis, Jerusalem District, 9700, Jerusalem, Palestine; Faculty of Medicine, Al-Quds University, University Street, Abu Dis, Jerusalem District, 9700, Jerusalem, Palestine; Department of Radiation Oncology, Augusta Victoria Hospital, East Jerusalem, Palestine

**Keywords:** colorectal cancer, glioblastoma multiforme, mismatch repair

## Abstract

Colorectal cancer (CRC) is the third most commonly diagnosed and leading cause of death worldwide. On the other hand, glioblastoma multiforme (GBM) is the most prevalent and aggressive primary malignant brain tumor in adults. Inherited diseases of DNA mismatch repair (MMR) can cause multiple cancers in the same patient including CRC and GBM. In this study, we report a 59-year-old woman presented with fatigue, constipation, abdominal distention, perianal pain, right-sided arm weakness, and personality changes. After investigations, it was diagnosed that sporadic metastatic CRC and GBM occurred simultaneously in the same patient, which was confirmed by colonoscopy, biopsy, imaging, and molecular testing. As the treatment of two cancers in the same patient is unique and complex, the absence of guidelines for such cases was discussed in a multidisciplinary tumor board including surgeons, medical, and radiation oncologists.

## Introduction

Glioblastoma multiforme (GBM) is the most common malignant brain tumor in adults, and the overall median of survival is less than 2 years under the standard of care treatment. Colorectal cancer (CRC) is the third most frequently diagnosed and leading cause of death in the developed world. CRC and GBM might be associated with some mismatch repair (MMR) mutations that might be inherited with Lynch and Turcot syndrome or sporadic. Mostly, these tumors come isolated, and it is very rare to have both of the tumors occurring in one patient simultaneously. In this study, our patient has both of the tumors without any genetic relations. So, in this study, we discuss the course of both diseases that the patient went through, the clinical findings, and how they responded to treatment [1].

## Case presentation

**Figure 1:**
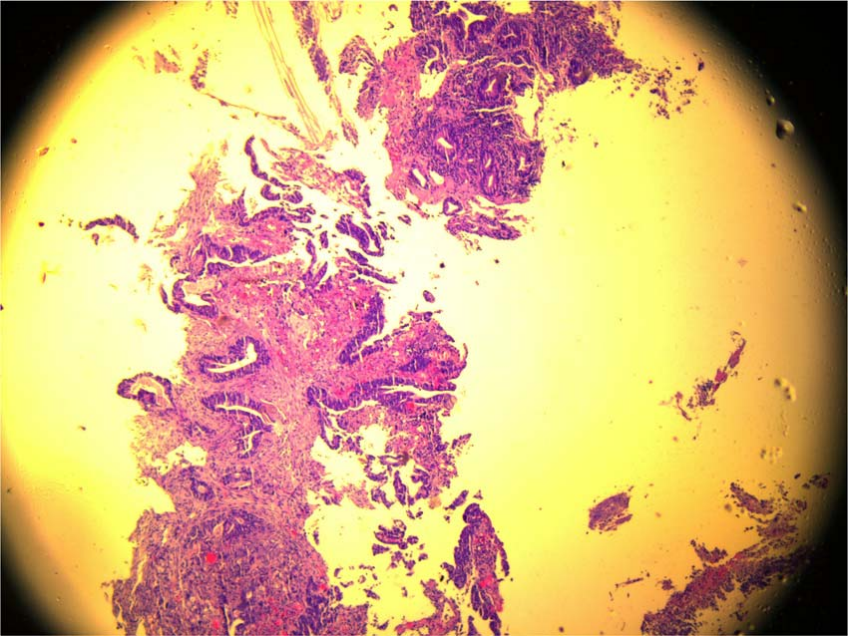
Microscopic view (×10) of pathology slide of colorectal cancer.

A 59-year-old female patient, married with no offspring. Surgical history includes cholecystectomy (2017). Her brother died due to lymphoma; otherwise, there is no family history of cancer. She presented complaining of chronic constipation, weight loss, abdominal distention, and perianal pain. Colonoscopy revealed a 5-cm mass extended from the anus. Biopsy revealed invasive, moderately differentiated rectal adenocarcinoma (Fig. 1).

**Figure 2:**
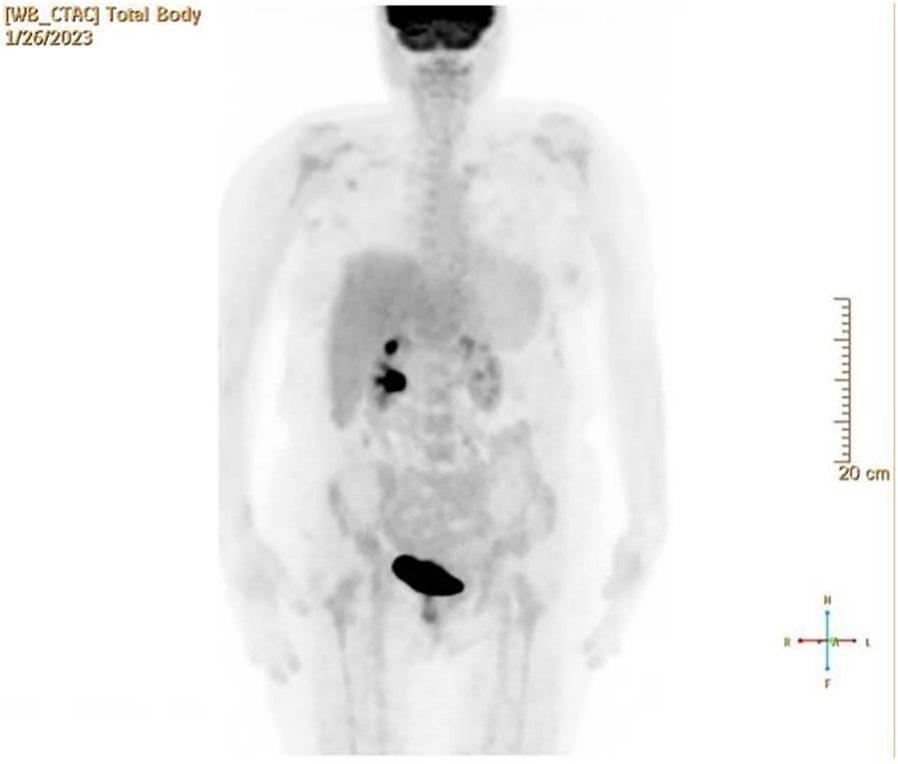
PET scan revealed multiple positive lymph nodes but no metastatic lesions in chest or abdominal organs or bone.

**Figure 3:**
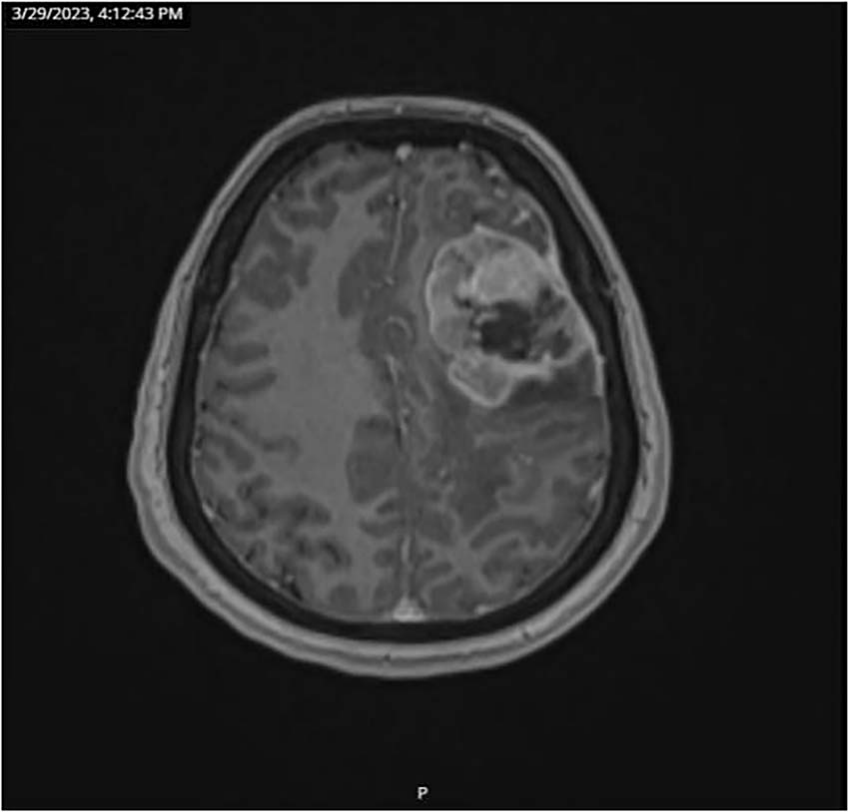
Brain MRI with gadolinium injection on 29 March 2023.

**Figure 4:**
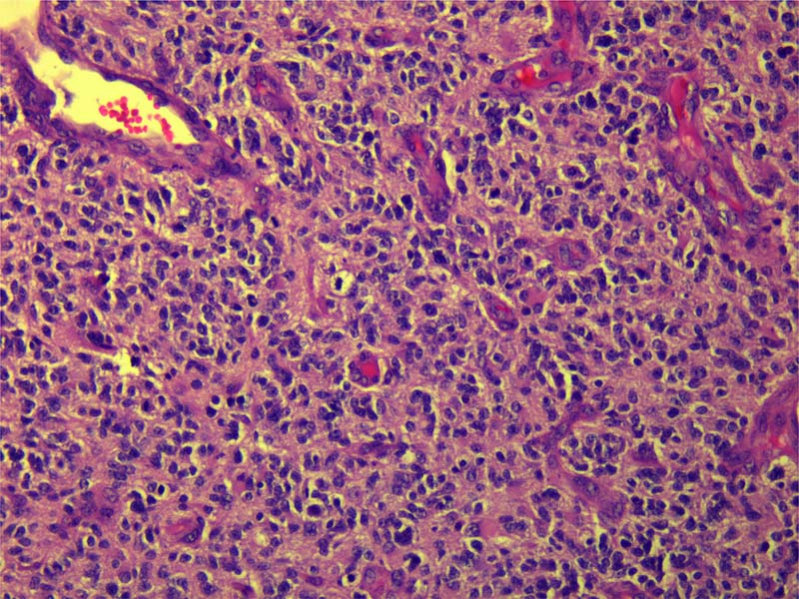
Microscopic view (×20) of pathology slide of GBM.

CT and PET scans revealed multiple positive lymph nodes, but metastatic lesions were not found in chest, abdominal organs, or bone (Fig. 2). Genetic and molecular tests revealed wild rat associated sarcoma, wild BRAF, proficient MMR, and negative HER-2. Tumor markers were positive. The patient started chemotherapy, which included CAPOX, cetuximab, and FOLFOX. PET scan was done for re-evaluation, and marked partial disease response was observed with currently no evidence of disease. After that, the patient started to experience reversible amnesia, bizarre behavior followed by right side upper limb weakness, hyperreflexia on the right side, positive Babinski sign on the left, and positive Hoffman sign bilaterally. Brain MRI was done, which revealed a left fronto-parietal extra-axial space-occupying lesion with vasogenic edema and midline shift (Fig. 3). Patient underwent brain lesion resection through left fronto-parietal craniotomy. The pathology revealed GBM (Fig. 4). IHC stain of neoplastic cells showed wild IDH and positive for GFAP, and 30% of the neoplastic cells showed positive for Ki67.

Follow-up CT scan revealed stable disease. Due to logistic issues, the patient was continued on FOLFOX + cetuximab, and then she was started on brain chemo-radiotherapy with temozolomide. Follow-up imaging showed disease progression according to RECIST criteria (new liver and lung metastatic lesions). So, the patient was started on systemic chemotherapy temozolomide-Irinotecan. Next follow-up showed mild disease progression. In general, the patient was having a stable course of GBM; however, with disease progression of CRC, she was started on temozolomide and planned for brain MRI to start oxaliplatin + cetuximab for six cycles then follow-up CT scan and tumor markers.

## Discussion

Hereditary germline mutations in MMR lead to a risk of 60%–70% CRC in men and 30%–40% in women [2]. 12.5% results from sporadic cases and 10% of all CRC are hereditary, including Turcot syndrome [2]. MMR deficiency brain tumors are very rare around 2% including GBM [3].

CRC is the second most common cancer to be diagnosed (10% of all cancers diagnosed) in women and third in men and ranks fourth in terms of cancer-related mortality (about 25% lower in women). Developed countries have a higher incidence, with a lifetime incidence of 5% [4].

On the other hand, the most prevalent and aggressive primary brain tumor in adults is called glioblastoma (less than 1% are hereditary). It has a low annual incidence of 3.1 per 100 000. White people are more impacted than Black people (2:1), and males are affected more than females (1.6:1) [5].

CRC risk factors are sporadic, environmental, and hereditary. Including male sex, increasing age, long-standing IBD, and colonic polyp, previous CRC shows a strong association with disease incidence and requires surveillance [6]. Hereditary risk factors are three times higher (20% of all patients) [7]. Moreover, modifiable environmental factors include smoking, excessive alcohol intake, increased body weight, type 2 DM, processed, and red meat. *Bacteroides fragilis* and *Fusobacterium nucleatum* are bacterial infections that increase the risk for CRC [6]. A family history of GBM is twofold higher in disease incidence. The radiation dose of diagnostic scans, head injury, and smoking do not suffice as risk factors. In conclusion, the only known external risk factor is exposure to ionizing radiation to the brain [5].

DNA MMR is a highly conserved biological mechanism that is essential for genomic stability. It corrects frameshift mutations in microsatellites and mismatched nucleotides. Impaired function leads to hereditary and familial cancers [8]. Proteins like MLH1, PMS2, MSH2, MSH6, PMS1, MSH3, MLH3, and Exo1 are responsible for the identification and repair of mismatched bases [2]. Hereditary lynch syndrome is an autosomal dominant disorder caused by mutations in MLH1 and MSH2. It quadruples the risk of high-grade gliomas [2, 3]. GBM can develop from a secondary brain tumor or arises de novo. GBMs derived from glial cells [1]. There are four transcriptional subclasses: classical, mesenchymal, proneural, and neural. Chromosome 7 amplifications, chromosome 10 deletions, EGFR amplification, EGFR mutations, and Ink4a/ARF locus deletion are observed in the classical type. The mesenchymal subtype exhibits high expression levels of MET, CHI3L1, and factor-κB and TNF pathways genes, and NF1 mutation/deletion. Proneural, lower-grade gliomas, and secondary glioblastomas share the same gene mutation expression of IDH1 and TP53 and changes in PDGFRA [5, 9].

Diagnosis of GBM occurs after new-onset seizure or neurology deficit. Histopathology shows necrosis and microvascular proliferation, high mitotic rates, anaplasia, and invasiveness. Clinical diagnosis includes brain imaging. Immunohistochemistry markers antibody selectively detects mutant IDH1R132H [5]. However, CRC is asymptomatic until it reaches an advanced stage. Investigations by colonoscopy are the method of choice [6].

Early surgical removal of the tumor is the most positive prognostic treatment for both diseases. It enhances the success rate in patients’ treatment. Maximally safe surgical resection, radiation therapy, and concurrent/adjuvant temozolomide are important in the management of GBM. Usage of 5-aminolevulinic acid or intraoperative imaging by ultrasound/MRI helps to obtain optimal resection [5]. On the other hand, CRC could be resected locally with no metastases. Treatment depends on the location of the tumor and the TNM staging system through the total mesorectal oncological approach, chemotherapy, and biologics. Perforated/obstructed colon cancer is treated through colostomy or endoscopic stenting [1].

Early detection prevents cancer progression and improves prognosis. A colonoscopy screening is suggested for people over the age of 45 for CRC detection. The 5-year survival rate exceeds 60% (USA) and 40% in developed countries [4]. Lifestyle modifications are ineffective in averting gliomas. Age has an inverse relationship with survival; after 5 years, 5% of all glioblastoma patients are still alive and drop to 2% for patients 65 years of age or older [5]. The survival rate of sporadic GBM is around 15 months, while GBM Turcot syndrome is around 27 months. Finally, symptomatic GBM and CRC have worse prognosis, management difficulties, and shorter survival rate [10].

## Conclusion

The co-occurrence of GBM with CRC is a rare and complex clinical presentation that underscores the critical importance of accurate diagnosis and diligent follow-up. In such cases, a thorough diagnostic process, including advanced imaging techniques and precise biopsy, is paramount to establish a definitive diagnosis. This enables a tailored treatment plan that can address these unique challenges. Moreover, in the context of GBM, a disease known for its aggressive nature and poor outcome, a robust follow-up is a cornerstone of patient care that can lead to improved survival rates and better management of these complex conditions.

## Data Availability

The data used to support the findings of this study are included within the article.
